# HGSM-YOLO: A Small-Lesion-Oriented Lightweight YOLO11n Framework for Citrus Leaf Disease Detection

**DOI:** 10.3390/s26175345

**Published:** 2026-08-24

**Authors:** Rui Zheng, Jing Zhao, Xinwei Wang, Feng Wang

**Affiliations:** School of Electronic Information, Xijing University, Xi’an 710123, China; 2508540606043@stu.xijing.edu.cn (R.Z.); 2508540606053@stu.xijing.edu.cn (X.W.); wangfengisn@163.com (F.W.)

**Keywords:** citrus leaf disease detection, YOLO11n, efficient object detection, HetConv, GSConv, slim neck, MSDA, multi-scale dilated attention, precision agriculture

## Abstract

Accurate and rapid detection of citrus leaf diseases is important for early diagnosis, precision orchard management, and the reduction of economic losses in citrus production. Automatic detection remains difficult because early lesions are often small and irregular. Several disease categories also share similar visual appearances, and localization is easily affected by veins, shadows, and cluttered backgrounds. To address these task-specific challenges, we propose HGSM-YOLO, where HGSM denotes the coordinated use of heterogeneous convolution, a GSConv-based slim neck, and multi-scale dilated local attention. The framework is built on YOLO11n because its 2.59 M-parameter and 6.4 GFLOP design provides a stringent compact baseline for edge-oriented improvement. The method follows a hierarchical design: C3k2-HetConv preserves lesion edges and local morphology in the backbone; the GSConv-based slim neck reduces part of the feature fusion cost; and an MSDA module in the high-resolution P3 branch enhances the context of small lesions. Following model selection on the validation split, the final locked models were evaluated once on the held-out test split, with HGSM-YOLO reaching 77.5% precision, 66.8% recall, 71.7% F1-score, 70.8% mAP@0.5, and 44.2% mAP@0.5:0.95, compared with 68.5%, 61.5%, 64.8%, 66.0%, and 40.2% for YOLO11n. A stratified outer five-fold cross-validation further yields 71.0% ± 1.4% mAP@0.5 and 44.4% ± 1.1% mAP@0.5:0.95 for HGSM-YOLO, versus 65.9% ± 1.1% and 40.2% ± 0.9% for YOLO11n. On the independent 1871-image citrus-leaf-disease-2 dataset, retraining under the same protocol gives 94.4% mAP@0.5 for HGSM-YOLO versus 92.2% for YOLO11n and 93.1% for the public Roboflow YOLOv11 reference model. The complete HGSM-YOLO architecture uses 7.2 GFLOPs, 2.82 M parameters, and runs at 90.9 FPS on the RTX 4090, compared with 6.4 GFLOPs, 2.59 M parameters, and 110.1 FPS for the baseline. Thus, the contribution provides a recall- and localization-oriented accuracy–efficiency trade-off rather than universal superiority in every individual metric.

## 1. Introduction

Citrus is one of the most widely cultivated fruit crops worldwide and carries high nutritional and economic value. During production, leaf diseases such as black spot, canker, Huanglongbing, and melanose lower photosynthetic efficiency, weaken tree vigor, and ultimately reduce fruit yield and quality. Early and accurate detection of these diseases is important for orchard management, disease warning, reduced pesticide use, and the broader goal of precision agriculture.

Citrus leaf disease identification still depends heavily on manual inspection and expert judgment. Manual diagnosis works in small orchards but is slow, subjective, and hard to scale to large production areas. In natural orchards, leaf images are further affected by changing illumination, leaf overlap, shooting angle, branch occlusion, cluttered backgrounds, and varying disease stages. Detection becomes harder still when lesions are small, their boundaries are blurred, and their colors resemble leaf veins or shadows.

Traditional image processing and machine learning methods usually rely on handcrafted features such as color, texture, shape, edge, and segmentation descriptors. These methods are computationally simple and perform well under controlled conditions, but their accuracy is unstable when the background is complex or illumination varies strongly. Because their features are manually defined, they generalize poorly across different orchards, cameras, and disease stages.

With the rapid progress of deep learning, convolutional neural networks and object detection algorithms have been widely applied to agricultural image analysis [1]. Unlike classification models, detectors identify disease categories and locate diseased regions at the same time, which suits practical disease monitoring and precision spraying. Two-stage detectors achieve high accuracy but have complex structures and slow inference [2]. Single-stage detectors such as the YOLO series regress categories and bounding boxes directly, which fits real-time agricultural use [3]. The YOLO family has continued to evolve through efficient real-time variants such as YOLOv7 [4]. Recent work on citrus and other crop disease detection has likewise adopted lightweight detectors and attention-based enhancement [5,6,7,8,9,10,11,12,13,14,15,16,17,18,19,20,21].

Existing agricultural detection studies can be grouped into three main design strategies, but each has a limitation that is important for lesion-level citrus detection. First, lightweight structural redesign reduces parameters or feature fusion cost. GSConv/slim neck provides an efficient neck design [22], and recent citrus or crop detectors employ lightweight backbones, pruning-oriented structures, or compact YOLO variants [5,8,9,10,11,21]. However, aggressive lightweighting can weaken the high-resolution texture cues needed for tiny spots and irregular lesion boundaries; many studies also report only aggregate mAP and do not verify whether small lesions benefit more than medium or large objects.

Second, attention-based methods improve contextual discrimination. DilateFormer shows that dilated local attention can combine multiple receptive fields [23], and agricultural detectors have introduced early attention, residual attention, and other feature-enhancement mechanisms [6,14,15,18]. Their limitation is that generic attention can add computation without identifying which feature level actually contains the critical lesion detail. For citrus disease, placing an attention module after substantial downsampling may be too late to recover information already lost.

Third, multi-scale fusion is widely used to strengthen small target detection [16,18]. Although these methods improve cross-scale representation, feature pyramid fusion can become a computational bottleneck itself, and the literature often does not jointly examine split sensitivity, small object metrics, external dataset transferability, or edge device speed. Literature reviews of plant disease detection similarly identify a shift toward real-time field recognition but note persistent challenges from limited datasets, clutter, illumination changes, and domain variation [12,13].

Recent YOLO11-based plant disease studies demonstrate that the YOLO family provides a competitive modern platform for agricultural detection [11,14,17,18]. Nevertheless, the unresolved question is not simply whether another module can raise an aggregate metric; a more specific gap concerns how a detector working under a compact edge budget can preserve fine lesion texture before downsampling, perform efficient cross-scale fusion, and enhance the high-resolution branch while also directly verifying that the gains are both robust to data partitioning and largest for small lesions. This gap motivates both the architecture and the revised evaluation protocol in this study.

In answering the above question, we select YOLO11n as the baseline for three reasons. First, it is the smallest YOLO11 variant, and so imposes a meaningful edge-oriented resource constraint. Second, its P3/P4/P5 feature hierarchy exposes clear insertion points for texture preservation, fusion redesign, and high-resolution attention. Finally, its compact configuration with 2.59 M parameters and 6.4 GFLOPs leaves limited spare capacity, making any accuracy gain under modest additional cost easier to interpret. We do not assume that YOLO11n has the highest precision among all compact YOLO models; instead, it serves as a controlled modern backbone for studying whether task-specific modifications improve the precision–recall and localization balance.

From this analysis, we formulate three testable design hypotheses. H1: Preserving heterogeneous spatial kernels in the backbone should improve local representation of lesion texture without replacing all spatial kernels with pointwise convolutions. H2: Replacing the conventional fusion path with a GSConv-based slim neck should reduce the local neck burden while maintaining cross-scale information flow. H3: Adding multi-scale dilated local attention specifically to P3 should improve recall, F1-score, and localization mAP by combining fine texture with surrounding context; therefore, the complete framework should improve multi-metric detection quality across held-out, cross-validation, and external-dataset evaluations, even if another compact detector retains a higher precision at a particular operating point. To address the stated problems, we propose HGSM-YOLO, a small lesion-oriented accuracy–efficiency framework for citrus leaf disease detection. The design is guided by three task requirements rather than by a simple accumulation of existing operators: preserving fine lesion texture in the backbone, strengthening cross-scale feature transmission in the neck, and increasing recall of subtle lesions in the high-resolution detection branch. Our main contributions are as follows:

(1) We design a C3k2-HetConv spatial texture extraction block for citrus lesion features. By embedding heterogeneous convolution into the convolution-intensive branch of C3k2, the block retains part of the 3×3 spatial kernels for lesion edges, spots, and irregular morphology while using 1×1 kernels to reduce redundant channel computation.
(2) We construct a GSConv-based slim neck for efficient cross-scale fusion. The neck is redesigned as the main efficiency bottleneck: GSConv and VoVGSCSPC are used to transmit shallow texture and deep semantic features between P3–P5, thereby improving multi-scale lesion recognition without making the backbone too light.
(3) We introduce an MSDA-enhanced P3 branch for small and weak lesions. Different attention heads use different dilation rates, enabling the high-resolution branch to combine immediate lesion texture with surrounding leaf context and reducing background interference and missed detections.
(4) We strengthen the experimental protocol by separating validation-based model selection from the final held-out test evaluation, adding fixed-split multi-seed statistics, stratified outer five-fold cross-validation, size-stratified lesion metrics, evaluation on the independent 1871-image citrus-leaf-disease-2 dataset, and real deployment tests for embedded devices. Together, these five aspects test sensitivity to the training/testing partition, while the external retraining experiment evaluates transferability under a different six-class citrus dataset which distinguishes retraining from zero-shot generalization.

## 2. The YOLO11n Baseline

YOLO11 is a recent real-time detector released by Ultralytics. It keeps the single-stage design of the YOLO family while refining the backbone, feature fusion, and detection head, achieving a strong balance among accuracy, inference speed, and parameter scale. Unlike two-stage detectors, it unifies localization and classification in an end-to-end network and supports tasks such as detection, instance segmentation, classification, pose estimation, and oriented detection [24].

We adopt YOLO11n, the lightweight variant of the series, as our baseline. With compound scaling parameters (depth = 0.50, width = 0.25, and max_channels = 1024), the model has about 181 layers, 2.59 M parameters, and 6.4 GFLOPs. Its small size and fast inference make it well suited to edge deployment in agricultural disease detection, where both detection speed and model size are constrained.

YOLO11n consists of a backbone, a neck, and a head. The backbone extracts multi-level features: stride-2 convolutions progressively halve the spatial resolution and expand the channels, producing the P1/2 to P5/32 representations, while C3k2 modules at each stage refine edges, textures, shapes, and local structure. At the end of the backbone, SPPF enlarges the receptive field by aggregating multi-scale context and C2PSA uses attention to emphasize key regions and informative channels. The neck follows an FPN/PAN-style scheme [25,26] in which deep semantic features are upsampled and concatenated with shallow features of matching scale, then fused by C3k2 modules and passed back down through downsampling and further concatenation. This top-down and bottom-up flow preserves both shallow spatial detail and deep semantics, improving detection across object sizes.

The detection head produces bounding boxes, class probabilities, and confidence from the three neck outputs P3/8, P4/16, and P5/32. The high-resolution P3 map carries rich spatial detail for small objects, P4 balances semantics and detail for medium objects, and P5 offers the largest receptive field and strongest semantics for large objects. For each candidate position, a bounding box regression branch (including DFL-based distribution) and a category branch output box and class predictions; non-maximum suppression then yields the final detections in a single forward pass. In the official configuration, this corresponds to detecting (P3, P4, P5) over layers 16, 19, and 22.

This three-scale head is well matched to the task of citrus leaf disease detection, as lesions vary widely in scale, occupy small areas, and differ only subtly between classes. Joint prediction across P3, P4, and P5 strengthens detection of small and medium lesions while also adapting to different growth stages and disease morphologies. Because YOLO11n combines a lightweight design, real-time speed, and strong multi-scale detection, we use it as the baseline and improve it for the specific problems of small targets, complex textures, and class similarity in agricultural disease detection.

## 3. The Proposed HGSM-YOLO Method

Ultralytics’ YOLO11 offers variants from YOLO11n to YOLO11x, with the different variants trading parameters and computation for accuracy. We choose YOLO11n not because it necessarily dominates every accuracy metric but because it is the most constrained member of the family and as such provides a stringent edge-oriented baseline. Its compact resource budget makes it possible to judge whether task-specific changes improve lesion detection without simply scaling the network. The baseline also exposes three feature stages that correspond directly to our diagnosed bottlenecks: local texture can be protected in the backbone, cross-scale transfer can be redesigned in the neck, and the high-resolution P3 branch can be enhanced before detection.

On this basis, we propose HGSM-YOLO. The acronym HGSM summarizes the three coordinated components: H denotes heterogeneous convolution in C3k2-HetConv, GS denotes the GSConv-based slim neck, and M denotes multi-scale dilated local attention (MSDA). Therefore, the name describes a structured feature pathway rather than an arbitrary collection of modules.

**Figure 1:**
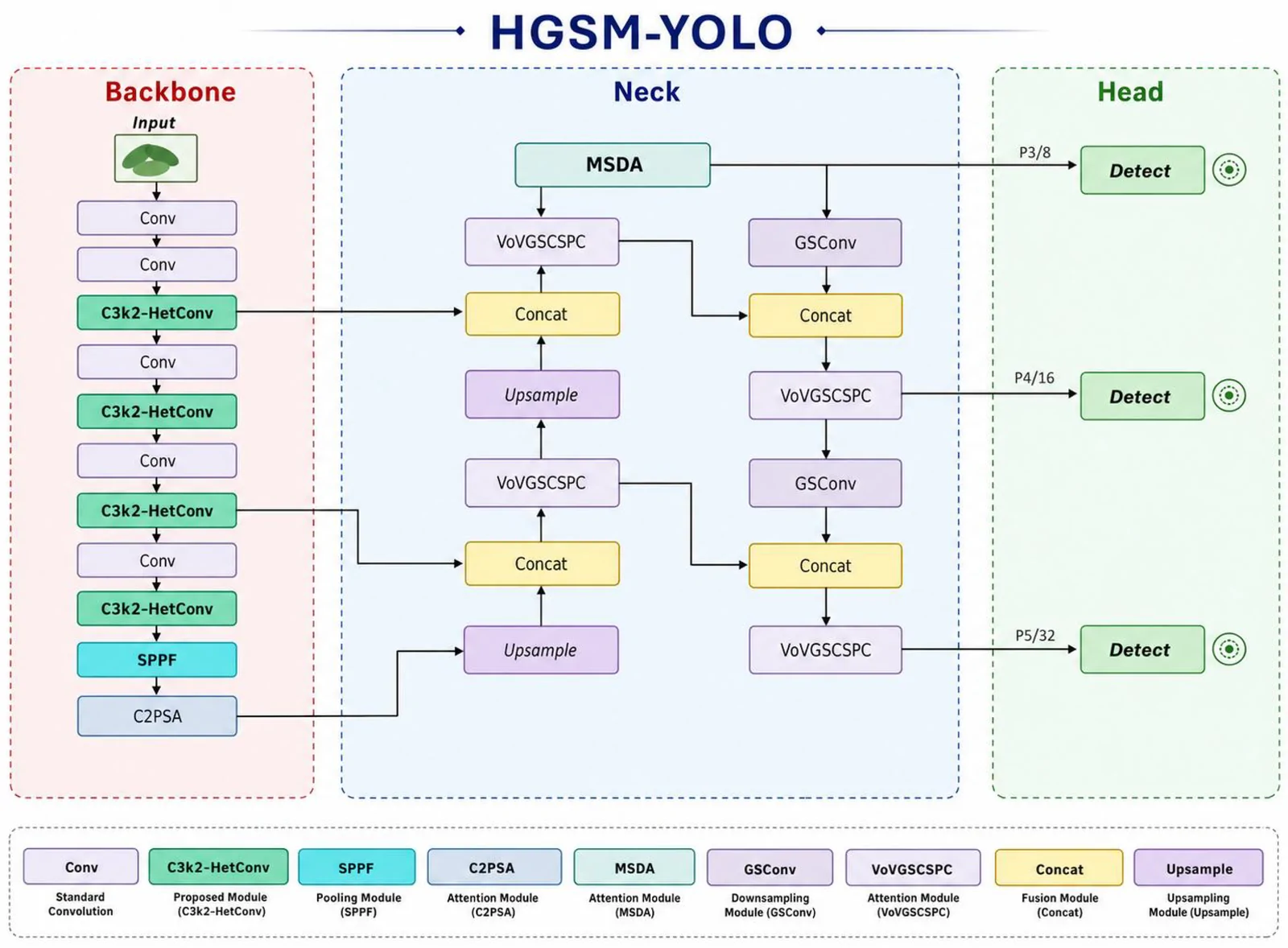
Overall architecture of HGSM-YOLO. The C3k2-HetConv module is used in the backbone for efficient feature extraction, the GSConv-based slim neck performs multi-scale feature fusion, and the MSDA module is embedded in the high-resolution P3 branch before the three-scale detection head.

The theoretical argument follows the order in which lesion information is processed. Small citrus lesions first require preservation of local edges, spots, and irregular morphology before repeated downsampling; C3k2-HetConv addresses this stage by retaining selected 3×3 spatial kernels while using cheaper 1×1 channel mappings. The resulting shallow and deep features then need cross-scale exchange; the GSConv-based slim neck targets this fusion bottleneck and reduces part of its computational cost. Finally, weak lesions in P3 require context to distinguish disease texture from veins, shadows, and healthy tissue; MSDA uses multiple dilation rates at this high-resolution stage. Thus, the modules are placed according to three successive failure modes, corresponding to texture loss, inefficient fusion, and insufficient small lesion context, which leads directly to hypotheses H1–H3 as stated in the Introduction. Figure 1 presents the overall HGSM-YOLO architecture and the placement of its three coordinated components.

### 3.1. C3k2-HetConv Module

To cut redundant computation in YOLO11n feature extraction while preserving the model’s ability to represent the edges, textures, and irregular shapes of citrus lesions, we introduce heterogeneous kernel convolution (HetConv) into the C3k2 structure, forming a C3k2-HetConv module [27]. HetConv applies kernels of different spatial sizes within one filter: some input channels use K×K kernels and the rest use 1×1 kernels. This combination lowers the parameter count and computational cost of a convolutional layer while retaining local spatial modeling.

**Figure 2:**
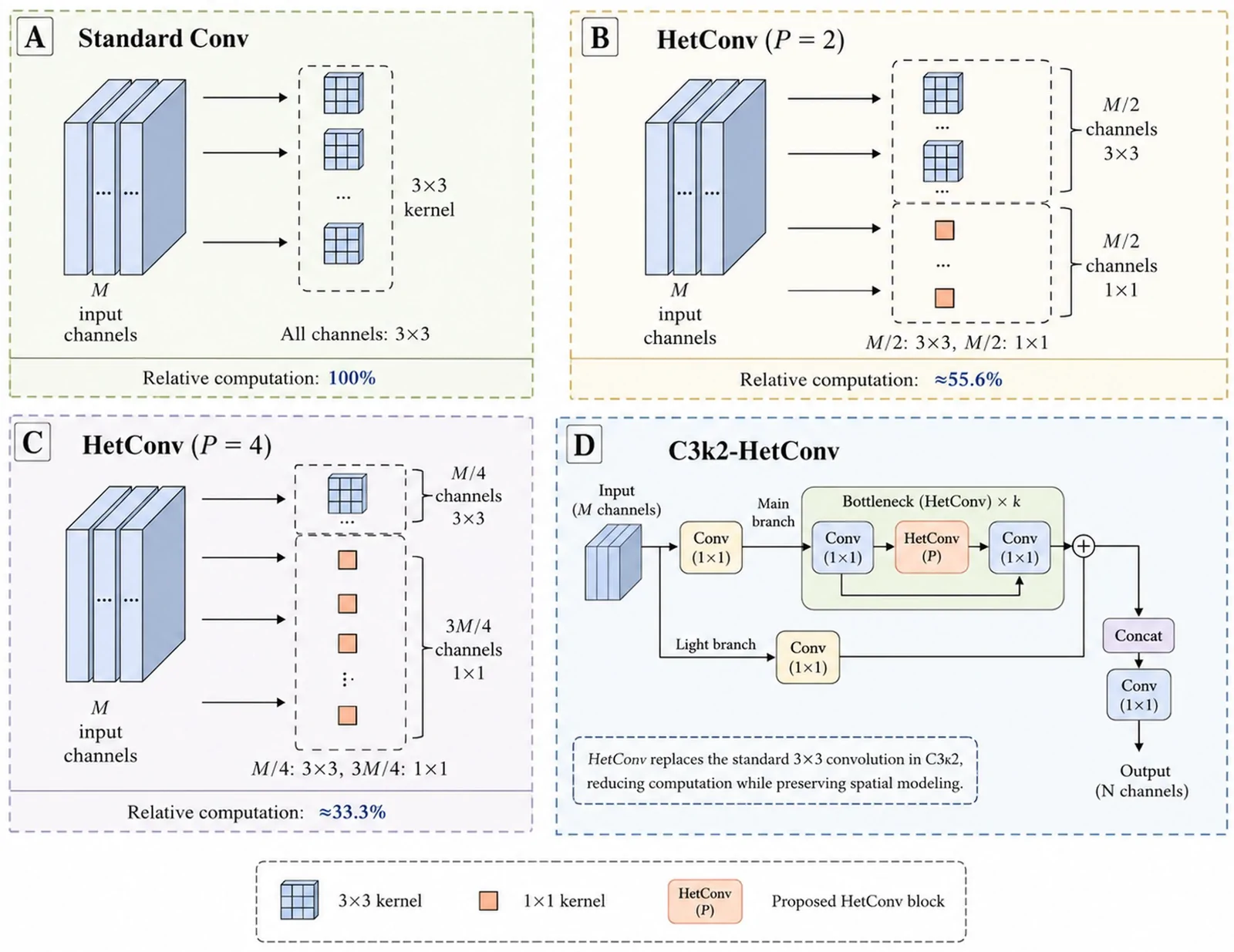
Mechanism of the C3k2-HetConv module. (**A**) Standard convolution applies 3×3 kernels to all input channels. (**B**,**C**) HetConv mixes 3×3 and 1×1 kernels within one filter, with the share of 3×3 kernels controlled by the ratio *P* (P=2 and P=4, respectively). (**D**) The C3k2-HetConv module integrates HetConv into the convolution-intensive branch of C3k2.

As shown in Figure 2, a standard filter applies 3×3 kernels to all *M* input channels, so every channel undergoes full spatial convolution. HetConv instead mixes 3×3 and 1×1 kernels within the filter. With ratio P=2, about half the channels use 3×3 kernels and the rest use 1×1 kernels; with P=4, only about a quarter use 3×3 kernels. As *P* grows, the share of 1×1 kernels rises and computation drops further. Crucially, HetConv does not replace 3×3 convolution outright but keeps a fraction of spatial kernels in each filter, balancing spatial feature extraction against the lightweight budget.

Let the input be $D_{i}\times D_{i}\times M$ and the output $D_{o}\times D_{o}\times N$, where *M* and *N* are the input and output channel counts and the kernel size is K×K. The cost of a standard convolutional layer, expressed as multiply–accumulate operations (MACs), is

(1) $$F_{\mathrm{LS}}=D_{o}\times D_{o}\times M\times N\times K\times K.$$

Because standard convolution performs full K×K convolution between all input and output channels, its cost grows quickly with channel count and kernel size. For HetConv with ratio *P*, only M/P input channels use K×K kernels and the remaining M−M/P channels use 1×1 kernels, giving

(2) $$F_{\mathrm{LHC}}=\frac{D_{o}\times D_{o}\times M\times N\times K\times K}{P}+D_{o}\times D_{o}\times N\times \left(M-\frac{M}{P}\right),$$

where the first term is the cost of the K×K kernels and the second that of the 1×1 kernels. By restricting expensive spatial convolution to part of the channels and using 1×1 convolution for channel mapping on the rest, HetConv lowers overall complexity. Comparing Equation (2) with Equation (1) gives the theoretical ratio

(3) $$R_{\mathrm{HetConv}}=\frac{F_{\mathrm{LHC}}}{F_{\mathrm{LS}}}=\frac{1}{P}+\frac{1-1/P}{K^{2}},$$

which depends only on *K* and *P*. For K=3:

(4) $$R_{\mathrm{HetConv}}=\frac{1}{2}+\frac{1-1/2}{9}\approx 0.556\;\left(P=2\right),$$

(5) $$R_{\mathrm{HetConv}}=\frac{1}{4}+\frac{1-1/4}{9}\approx 0.333\;\left(P=4\right),$$

i.e., about 55.6% and 33.3% of the standard convolution cost, respectively. Thus, for 3×3 convolution, HetConv markedly reduces computation while retaining part of the spatial modeling capacity.

In citrus leaf disease detection, lesions are small, irregular, and texturally distinct, making them easily confused with veins and background. Using only 1×1 convolution would cut computation but weaken perception of local spatial structure, whereas using only 3×3 convolution is costly. HetConv resolves this trade-off by keeping 3×3 kernels on some channels for lesion edges, spot textures, and local morphology and keeping 1×1 kernels on others for lightweight channel mapping. Therefore, we insert HetConv into the convolution-intensive branch of C3k2, replacing part of the standard 3×3 convolution with the heterogeneous combination, and apply C3k2-HetConv mainly in the backbone. This preserves multi-scale feature extraction for lesion edges, textures, and local shapes while improving module-level computational efficiency and deployment potential.

### 3.2. GSConv-Based Slim Neck

The neck fuses shallow detail features from the backbone with deep semantic features, which is key to detecting targets across scales. Citrus lesions range from small spots to large discolored areas, and are easily confused with veins, shadows, and background texture; thus, the model needs both low-level edge/texture/color cues and high-level semantics. However, the standard YOLO neck stacks many standard convolutions for channel transformation and fusion, inflating parameters and computation. We introduce GSConv and the slim neck into YOLO11n to cut cost while preserving multi-scale representation, consistent with prior work on lightweight necks for real-time detectors [22].

**Figure 3:**
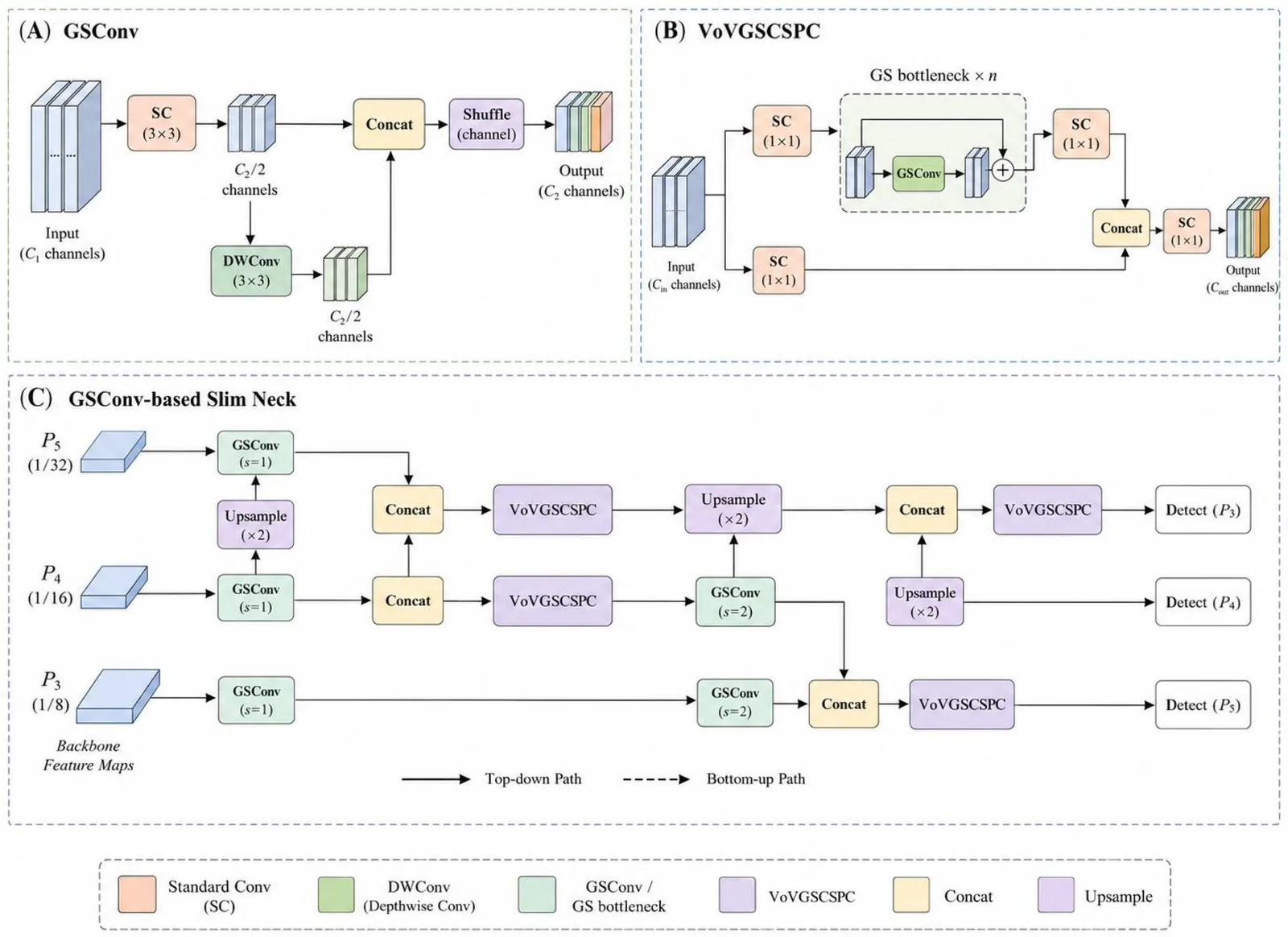
GSConv-based lightweight fusion structures. (**A**) GSConv combines standard convolution, depthwise separable convolution, and channel shuffle. (**B**) The VoVGSCSPC module aggregates GSConv units through cross-stage partial connections. (**C**) The GSConv-based slim neck performs multi-scale feature fusion with reduced computation.

GSConv combines standard convolution (SC), depthwise separable convolution (DSC), and channel shuffle. As shown in Figure 3A, the input *X* first passes through SC to produce $Y_{\mathrm{sc}}$ with half the output channels; $Y_{\mathrm{sc}}$ then feeds DSC to produce a complementary set $Y_{\mathrm{dsc}}$; finally, the two are concatenated along the channel dimension and reordered by a shuffle operation to give $Y_{\mathrm{out}}$. Figure 3 summarizes the GSConv operator, the VoVGSCSPC module, and the GSConv-based slim neck.

Let the input be $X\in R^{H\times W\times C_{1}}$ and the output $Y_{\mathrm{out}}\in R^{H\times W\times C_{2}}$, where *H* and *W* are the height and width, respectively, $C_{1}$ is the input channel count, and $C_{2}$ is the output channel count. GSConv is computed as follows:

(6) $$Y_{\mathrm{sc}}=\mathrm{SC}\left(X\right)$$

(7) $$Y_{\mathrm{dsc}}=\mathrm{DSC}\left(Y_{\mathrm{sc}}\right)$$

(8) $$Y_{\mathrm{cat}}=\mathrm{Concat}\left(Y_{\mathrm{sc}},Y_{\mathrm{dsc}}\right)$$

(9) $$Y_{\mathrm{out}}=\mathrm{Shuffle}\left(Y_{\mathrm{cat}}\right)$$

where $Y_{\mathrm{sc}},Y_{\mathrm{dsc}}\in R^{H\times W\times C_{2}/2}$ (so that $Y_{\mathrm{cat}}\in R^{H\times W\times C_{2}}$) and Shuffle(·) reorders the channels so the dense SC features and the lightweight DSC features interact fully.

For standard convolution with $C_{\mathrm{in}}$ input channels, $C_{\mathrm{out}}$ output channels, and kernel size K×K, the cost is

(10) $$F_{\mathrm{SC}}=H\times W\times C_{\mathrm{in}}\times C_{\mathrm{out}}\times K\times K.$$

Standard convolution couples spatial extraction with cross-channel fusion, which makes it expressive; however, its cost rises sharply with channel count, which is costly in the high-channel neck. Depthwise separable convolution is composed of a depthwise and a pointwise step [28], which decouples the two:

(11) $$F_{\mathrm{DSC}}=H\times W\times C_{\mathrm{in}}\times K\times K+H\times W\times C_{\mathrm{in}}\times C_{\mathrm{out}}.$$

DSC is far cheaper, yet when used alone it weakens cross-channel fusion. GSConv keeps half the output channels from SC and generates the other half through DSC. Its approximate cost is given below.

The following GSConv expressions likewise count MACs; their conversion to profiled FLOPs follows the convention defined in Equation (43):

(12) $$\begin{array}{cc} F_{\mathrm{GSConv}}= & H\times W\times C_{1}\times \left(C_{2}/2\right)\times K_{s}\times K_{s}\\ \; & +H\times W\times \left(C_{2}/2\right)\times K_{d}\times K_{d}\\ \; & +H\times W\times \left(C_{2}/2\right)\times \left(C_{2}/2\right) \end{array}$$

where $K_{s}$ and $K_{d}$ are the kernel sizes of the standard and depthwise convolutions; the three terms are the SC branch, DSC depthwise step, and DSC pointwise step. Mapping *X* directly to the output by standard convolution would instead cost

(13) $$F_{\mathrm{Standard}}=H\times W\times C_{1}\times C_{2}\times K\times K,$$

so the GSConv-to-standard ratio is:

(14) $$R_{\mathrm{GSConv}}=\frac{F_{\mathrm{GSConv}}}{F_{\mathrm{Standard}}}.$$

$R_{\mathrm{GSConv}}<1$ confirms that GSConv is lighter, since only part of the output is built by standard convolution. Channel shuffle, introduced in ShuffleNet [29], then interleaves the SC and DSC channels as defined in Equations (8) and (9). Here, [·] denotes channel concatenation. Shuffling adds no learnable parameters and negligible cost while compensating for the weak channel mixing of DSC.

We apply GSConv in the neck rather than the backbone. The early backbone extracts edges, colors, textures, and fine lesion cues, so an excessively lightweight design would harm the perception of small and irregular lesions. By contrast, the neck has low spatial resolution but high channel dimensions, where standard convolution is most costly. Thus, GSConv in the neck reduces fusion cost while having little impact on spatial detail.

To further strengthen fusion, we add the VoVGSCSPC module at the fusion nodes. Built on GSConv and cross-stage partial connections, it routes one branch through GSConv-based units for deep feature extraction and preserves the other through a cross-stage shortcut, then concatenates them. Compared with ordinary bottleneck or CSP structures, VoVGSCSPC fuses multi-scale features more efficiently, improving recognition of small lesions, irregular-edged lesions, and disease regions under background interference.

The slim neck reduces the proportion of standard convolutions in fusion. For YOLO11n features P3–P5, P3 has high resolution with rich edge/texture/color detail and P5 has strong semantics at low resolution. We fuse them with VoVGSCSPC and use GSConv on the key bottom-up downsampling path:

(15) $$F_{4}=\mathrm{VGS}\left(\mathrm{Concat}\left(\mathrm{Up}\left(B_{5}\right),B_{4}\right)\right)$$

(16) $$F_{3}=\mathrm{VGS}\left(\mathrm{Concat}\left(\mathrm{Up}\left(F_{4}\right),B_{3}\right)\right)$$

(17) $$F_{3}^{\prime }=A_{\mathrm{msda}}\left(F_{3}\right)$$

(18) $$N_{4}=\mathrm{VGS}\left(\mathrm{Concat}\left(\mathrm{GSConv}\left(F_{3}^{\prime }\right),F_{4}\right)\right)$$

(19) $$N_{5}=\mathrm{VGS}\left(\mathrm{Concat}\left(\mathrm{GSConv}\left(N_{4}\right),B_{5}\right)\right)$$

where Up(·), Concat(·), GSConv(·), and VGS(·) respectively denote upsampling, channel concatenation, lightweight convolution, and the VoVGSCSPC module, $F_{4}$ and $F_{3}$ are the top-down P4 and P3 features, $F_{3}^{\prime }$ is the P3 feature enhanced by MSDA, and $N_{4}$ and $N_{5}$ are the bottom-up P4 and P5 detection features. Thus, the deep semantics flow gradually to the high-resolution P3 branch, MSDA strengthens its perception of small lesions and complex textures, and the enhanced $F_{3}^{\prime }$ is downsampled by GSConv to feed the P4 and P5 branches, returning shallow detail to the mid- and high-level detectors.

For this task, the slim neck offers three benefits: the SC branch in GSConv preserves channel expressiveness to separate blackspot, canker, greening, melanose, and healthy classes; the DSC branch lowers computation in the neck for lightweight deployment; and channel shuffling fuses shallow texture with deep semantics across channels, improving multi-scale lesion recognition. Overall, GSConv + slim neck avoids the loss of fine lesion features from the backbone being too lightweight and removes the redundancy of repeated standard convolutions in the neck, supporting deployability by sustaining accuracy while reducing the cost of the neck subnetwork.

### 3.3. MSDA: Multi-Scale Dilated Local Attention Module

Attention guides the detector toward discriminative lesions while suppressing veins, shadows, and non-disease spots. Because citrus lesions vary in scale, have irregular boundaries, and sit in complex backgrounds, we introduce a multi-scale dilated local attention (MSDA) module inspired by the dilated attention of DilateFormer [23]. By splitting attention heads into groups with different dilation rates, MSDA models local context at several spatial scales and at low cost. Plain convolution has a receptive field bounded by kernel size, so enlarging it by stacking layers or large kernels is expensive; conversely, global self-attention is too costly on high-resolution maps. MSDA combines local window attention, dilated sampling, and multi-head parallel modeling to enlarge the effective receptive field while avoiding the cost of global attention.

Let the input be $X\in R^{H\times W\times C}$, where *H* and *W* are the height and width and *C* the number of channels. MSDA first generates query, key, and value features by linear mapping or 1×1 convolution:

(20) $$Q=XW_{Q},$$

(21) $$K=XW_{K},$$

(22) $$V=XW_{V}$$

where $W_{Q}$, $W_{K}$, and $W_{V}$ are the mapping weights while *Q*, *K*, and *V* are split into *h* heads along the channel dimension, each of dimension

(23) $$d_{h}=C/h.$$

The *m*-th head has $Q_{m}$, $K_{m}$, $V_{m}$ and a dilation rate $r_{m}$. For a k×k window with dilation *r*, the effective receptive field is

(24) $$K_{\mathrm{eff}}=k+\left(k-1\right)\times \left(r-1\right).$$

With k=3, this gives $K_{\mathrm{eff}}=3,5,7$ for r=1,2,3 (Equations (25)–(27)):

(25) $$K_{\mathrm{eff}}\left(r=1\right)=3,$$

(26) $$K_{\mathrm{eff}}\left(r=2\right)=5,$$

(27) $$K_{\mathrm{eff}}\left(r=3\right)=7.$$

Thus, a larger dilation rate widens the sampled region while keeping the number of window samples fixed. MSDA does not add tokens but can reach farther neighbors through dilated sampling, gaining receptive field at low cost. For a query at position (i,j), the dilated neighborhood is

(28) $$\Omega_{r}\left(i,j\right)=\left\{\;\left(i+r\Delta x,\;j+r\Delta y\right)|\Delta x,\Delta y\in \left[-\left(k-1\right)/2,\;\left(k-1\right)/2\right]\;\right\},$$

where *k* is the window size and Δx, Δy are offsets within it. Standard self-attention is

(29) $$\mathrm{Attention}\left(Q,K,V\right)=\mathrm{Softmax}\;\left(\frac{QK^{T}}{\sqrt{d}}\right)V,$$

where *d* is the feature dimension. Computed globally over all H×W tokens, its complexity is

(30) $$O_{\mathrm{global}}=O\;\left({\left(H\times W\right)}^{2}\times C\right),$$

which grows quadratically with the number of tokens and is impractical on high-resolution maps. MSDA instead restricts attention to the dilated neighborhood $\Omega_{r_{m}}\left(i,j\right)$ of each query. For the *m*-th head:

(31) $$A_{m}\left(i,j\right)=\mathrm{Softmax}\;\left(\frac{q_{m}\left(i,j\right)\;K_{m}{\left(\Omega_{r_{m}}\left(i,j\right)\right)}^{T}}{\sqrt{d_{h}}}\right),$$

(32) $$Y_{m}\left(i,j\right)=A_{m}\left(i,j\right)\;V_{m}\left(\Omega_{r_{m}}\left(i,j\right)\right),$$

where $q_{m}\left(i,j\right)$ is the query vector at (i,j), $K_{m}\left(\Omega_{r_{m}}\left(i,j\right)\right)$ and $V_{m}\left(\Omega_{r_{m}}\left(i,j\right)\right)$ are the key and value sets over the dilated neighborhood, $A_{m}\left(i,j\right)$ is the local attention weight, and $Y_{m}\left(i,j\right)$ is the head output. Because each query attends to only k×k samples, the MSDA complexity is

(33) $$O_{\mathrm{MSDA}}=O\;\left(H\times W\times k^{2}\times C\right),$$

which is linear in H×W rather than quadratic, making MSDA suitable for high-resolution branches. With dilation set $R=\{r_{1},r_{2},\ldots ,r_{h}\}$, the multi-head output is

(34) $$Y_{\mathrm{MSDA}}=\mathrm{Concat}\left(Y_{1},Y_{2},\ldots ,Y_{h}\right)\;W_{O},$$

where $W_{O}$ is the output mapping weight that fuses local texture, lesion edges, and longer-range context across channels.

By default, MSDA uses a 3×3 local window with dilation rates r=1,2,3. At r=1 attention focuses on the immediate neighborhood, capturing lesion edges, small spots, and fine textures; at r=2, the field expands to 5×5, capturing the relation between a lesion and adjacent tissue; and at r=3 it reaches 7×7, capturing larger disease-spread regions and background context. The three rates jointly cover local detail and multi-scale semantics.

**Figure 4:**
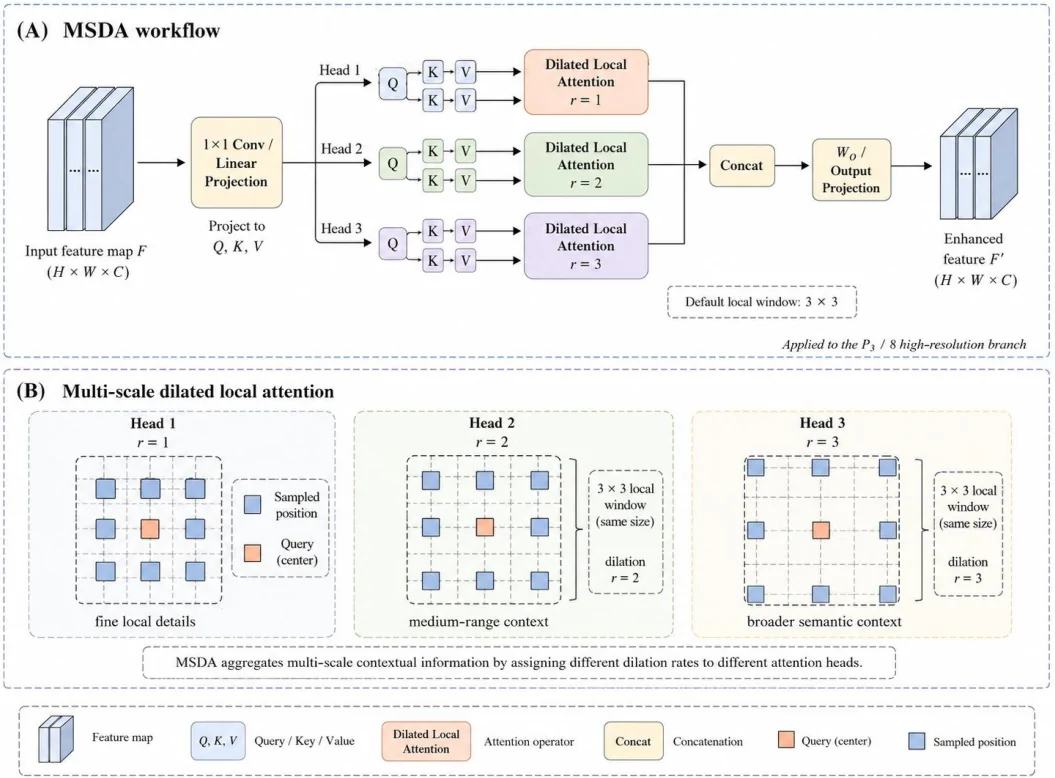
The MSDA multi-scale dilated local attention module. (**A**) In the workflow, query/key/value features are split into heads and each head performs local window attention with its own dilation rate. (**B**) Dilated local windows for dilation rates r=1,2,3, giving effective receptive fields of 3×3, 5×5, and 7×7.

In HGSM-YOLO, MSDA is embedded in the high-resolution P3 branch of the neck to enhance the VoVGSCSPC-fused feature $F_{3}$ into $F_{3}^{\prime }$. This enhanced feature feeds the small-scale detection branch, and also feeds the bottom-up fusion of the P4 and P5 branches after GSConv downsampling. Thus, MSDA sharpens the perception of small lesions, fine textures, and irregular edges while supplying richer detail to the higher-level branches. The complete MSDA workflow and its dilated local windows are illustrated in Figure 4.

For citrus leaf disease detection, MSDA provides several advantages: local window attention keeps the cost low enough for high-resolution maps, preserving perception of small lesions; dilated sampling enlarges the effective receptive field without adding samples, covering more leaf structure and disease context; and multi-head modeling at different scales helps to distinguish blackspot, canker, greening, and melanose while suppressing veins, shadows, uneven illumination, and non-disease spots. Compared with global self-attention, MSDA is far cheaper, while compared with plain local attention it can reach a larger field through dilation. Embedding it in the P3 branch strengthens representation of subtle and small lesions under complex backgrounds while improving localization and recognition accuracy.

## 4. Experiments and Results

### 4.1. Experimental Configuration

All experiments were run on a remote server, with training, validation, and test evaluation performed server-side. The server used an NVIDIA GeForce RTX 4090 GPU (24 GB; NVIDIA Corporation, Santa Clara, CA, USA) and an AMD EPYC 7502 32-Core processor (Advanced Micro Devices, Inc., Santa Clara, CA, USA) running Ubuntu 22.04 with PyTorch 2.5.1, CUDA 12.2, and Python 3.10. Training and evaluation used the Ultralytics framework. Ultralytics 8.3.200 was used for the YOLOv8, YOLO11n, and HGSM-YOLO experiments. Because YOLO26 was introduced after that package release, YOLO26n was evaluated with its official supported implementation and the yolo26n.pt weight. A dedicated reproducibility table later in this section separates this model identity from the 8.3.200-based experiments rather than attributing every Ultralytics model to one package version.

The main models were trained for up to 200 epochs with batch size 32 and input size 640 × 640, using SGD on one GPU with eight data-loading workers and early stopping patience of 50. Model architecture, hyperparameters, and the operating confidence threshold were selected only from the training and validation splits. After these choices were frozen, the locked YOLO11n and HGSM-YOLO models were evaluated on the held-out test split for the headline results. A separate uniformly validation-split comparison of compact detectors is reported later and is not used as a test set result.The confidence threshold sweep and ablation study are explicitly reported on the validation split, and are not used as test set results.

The five-seed experiment uses the same fixed train/validation/test split for all runs; only weight initialization, data-loader shuffling, and stochastic training operations change with seed. The five seeds were {0,1,2,3,4}. Consequently, this experiment measures training stochasticity on a fixed split; it does not by itself establish independence from the chosen data split. Paired tests match YOLO11n and HGSM-YOLO runs by identical seed.

To assess dependence on the particular 8:1:1 split, we additionally perform a stratified outer five-fold cross-validation over all 1250 main-dataset images. Each outer fold holds approximately 250 images for testing. From the approximately 1000 remaining images, 10% are reserved internally for validation and the remainder are used for training; no image from an outer test fold participates in model selection. The same fold assignments, augmentation recipe, pretrained initialization, optimizer, and epoch budget are used for YOLO11n and HGSM-YOLO. Results are summarized across the five outer test folds as mean ± standard deviation.

All geometric and photometric augmentations are applied online to training images only. Validation and test images receive only deterministic resizing and normalization. The same augmentation recipe is used for YOLO11n, HGSM-YOLO, and the compact YOLO comparison models wherever the implementation supports the same operation. This prevents augmentation strength from becoming a hidden source of unfairness in the model comparison.

### 4.2. Dataset and Evaluation Metrics

We used version 1 of the public Citrus Leaves Diseases object detection dataset on Roboflow Universe [30]. The cited version contains 1250 images in five classes (blackspot, canker, greening, healthy, and melanose) and is distributed under the CC BY 4.0 license. The public version applies auto-orientation and resizing to 640 × 640 and reports no Roboflow-side augmentation. We downloaded the YOLO-format annotations and created one fixed 8:1:1 split (1000/125/125 images) for training, validation, and final testing. One fixed set of image identities was used by every reported model; the split policy and class/instance counts are reported below, and the corresponding filename manifests are retained with the experimental records for release with the code.

The training split is used for parameter learning; the validation split is used for early stopping, architecture/hyperparameter selection, and operating-point selection; and the test split is reserved for the final locked model evaluation. The five-seed analysis repeats training on this same split and quantifies stochastic training variation. Split sensitivity is assessed separately by the stratified outer five-fold cross-validation described above, so the two protocols isolate different sources of uncertainty.

An annotation audit is also reported explicitly because the “healthy” class can have a different semantic granularity from visible disease symptoms. Healthy boxes are treated as whole-leaf annotations, whereas the four disease classes are treated as symptomatic region annotations. To avoid mixing these sources of granularity in the small lesion claim, the size-stratified lesion analysis below is computed on the four disease classes only, while the standard five-class metrics retain the healthy class.

The fixed split manifests and per-class image/instance counts in Table 1 are used for all reported main dataset experiments.

**Figure 5:**
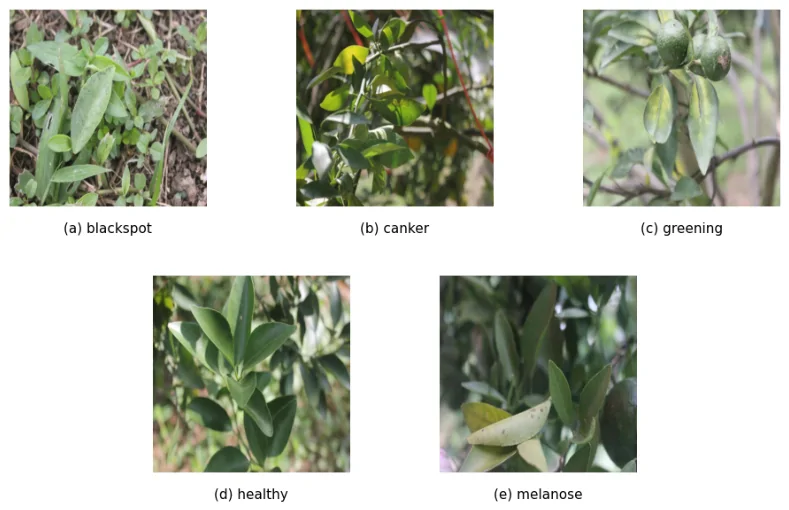
Representative samples of the five categories in the Citrus Leaves Diseases dataset: blackspot, canker, greening, healthy, and melanose.

Representative images from the five categories are shown in Figure 5.

The complete training and reproducibility settings are summarized in Table 2.

We evaluate detection with precision (P), recall (R), F1-score, mAP@0.5, and mAP@0.5:0.95, and evaluate efficiency with GFLOPs, model size, parameter count, and FPS.

Precision is the fraction of predicted positives that are correct; higher P means fewer false detections:

(35) $$P=\frac{\mathrm{TP}}{\mathrm{TP}+\mathrm{FP}}$$

where TP and FP are true and false positives. Recall is the fraction of true targets detected; higher R means fewer missed detections:

(36) $$R=\frac{\mathrm{TP}}{\mathrm{TP}+\mathrm{FN}}$$

where FN is the number of missed targets. F1-score is the harmonic mean of P and R, summarizing accuracy and completeness when the two are imbalanced:

(37) $$F1=\frac{2\;\mathrm{PR}}{P+R}.$$

AP is the area under the per-class precision–recall curve, approximated discretely:

(38) $$\mathrm{AP}=\int_{0}^{1}P\left(R\right)\;\mathrm{dR}\approx \sum_{n}\left(R_{n}-R_{n-1}\right)\;P_{n}$$

where $P_{n}$ and $R_{n}$ are the precision and recall along the full ranked prediction list as the score threshold is swept. Thus, AP and mAP represent curve-integrated metrics, and are not treated as metrics of one fixed operating confidence threshold in this study. mAP averages AP over all *C* classes (C=5 here):

(39) $$\mathrm{mAP}=\frac{1}{C}\sum_{c=1}^{C}{\mathrm{AP}}_{c}.$$

mAP@0.5 is the mean AP at IoU =0.5, where IoU measures the overlap between the predicted and ground-truth boxes:

(40) $$\mathrm{IoU}=\frac{A_{\mathrm{pred}}\cap A_{\mathrm{gt}}}{A_{\mathrm{pred}}\cup A_{\mathrm{gt}}},\;\mathrm{mAP}@0.5=\frac{1}{C}\sum_{c=1}^{C}{\mathrm{AP}}_{c}^{\;\mathrm{IoU}=0.5}$$

where $A_{\mathrm{pred}}$ and $A_{\mathrm{gt}}$ are the predicted and ground-truth box regions; this reflects detection under a loose localization criterion. mAP@0.5:0.95 averages mAP over IoU thresholds from 0.50 to 0.95 in steps of 0.05, imposing a stricter localization requirement:

(41) $$\mathrm{mAP}@0.5\;:\;0.95=\frac{1}{10}\sum_{t\in \{0.50,0.55,\ldots ,0.95\}}\mathrm{mAP}@t.$$

Params is the total number of learnable parameters (smaller is more lightweight):

(42) $$\mathrm{Params}=\sum_{l=1}^{L}N_{l},\;{\mathrm{Params}}_{\mathrm{conv}}=K_{h}\times K_{w}\times C_{\mathrm{in}}\times C_{\mathrm{out}}+C_{\mathrm{out}}$$

where *L* is the number of layers, $N_{l}$ denotes the learnable parameters in the *l*-th layer, $K_{h}$, $K_{w}$ are the kernel height and width, and $C_{\mathrm{in}}$, $C_{\mathrm{out}}$ are the input and output channels (the bias term $C_{\mathrm{out}}$ is omitted when unused). Section 3 uses MACs to express analytical layer cost because the relative ratios are unchanged by a common constant factor. The profiled values in the experimental tables are reported as GFLOPs, using the explicit convention that one MAC contains one multiplication and one addition and as such is counted as two floating-point operations:

(43) $$\mathrm{GFLOPs}=\frac{\mathrm{FLOPs}}{10^{9}},\;{\mathrm{FLOPs}}_{\mathrm{conv}}=2H_{\mathrm{out}}W_{\mathrm{out}}C_{\mathrm{out}}\frac{K_{h}K_{w}C_{\mathrm{in}}}{G}$$

where $H_{\mathrm{out}}$ and $W_{\mathrm{out}}$ are the output spatial size and *G* is the number of groups (G=1 for ordinary convolution). Thus, the reported 6.4 and 7.2 GFLOPs for YOLO11n and HGSM-YOLO, respectively, follow the same two-FLOPs-per-MAC convention. The MAC expressions in Section 3 are used for relative module comparisons, whereas all tabulated model profiles use Equation (43). Model size is the storage of the trained weights, estimable from the parameter count and per-parameter bytes:

(44) $$\mathrm{Model}\;\mathrm{Size}=\frac{\mathrm{File}\;\mathrm{Size}}{1024^{2}}\approx \frac{\mathrm{Params}\times B}{1024^{2}}$$

where file size is the bytes of the weight file and *B* is the bytes per parameter (4 under FP32). FPS is the number of images processed per second; for a per-image latency *t* (or $t_{\mathrm{ms}}$ in milliseconds):

(45) $$\mathrm{FPS}=\frac{N}{T}=\frac{1}{t}=\frac{1000}{t_{\mathrm{ms}}}$$

where *N* is the number of images and *T* is the total inference time. In short, P, R, and F1 capture false and missed detections; mAP@0.5 and mAP@0.5:0.95 assess recognition and localization; and GFLOPs, model size, params, and FPS measure efficiency and real-time capability.

### 4.3. Ablation Study

To verify the contribution of each component, we ran ablations under the same data, training, and evaluation settings. Table 3 reports the baseline YOLO11n and combinations of the MSDA, C3k2-HetConv, and slim neck on the validation split. Architecture decisions were made from this validation analysis before the final test evaluation was performed.

Adding MSDA alone improves the small lesion branch by raising recall from 0.620 to 0.636 and mAP@0.5:0.95 from 0.406 to 0.423. This trend supports the motivation that multi-scale dilated local attention helps to recover weak lesion cues while retaining localization quality. C3k2-HetConv mainly improves the discriminative quality of local texture features, increasing precision to 0.735 while maintaining a compact parameter scale. The slim neck reduces the computational cost of feature fusion, lowering GFLOPs from 6.4 to 5.8 and parameters from 2.59 M to 2.49 M while still improving mAP.

The full HGSM-YOLO obtains the best overall balance. Compared with YOLO11n, its precision, recall, F1-score, mAP@0.5, and mAP@0.5:0.95 increase by 9.1, 5.5, 7.2, 5.0, and 4.1 percentage points, respectively. The improvement in recall is particularly important for early disease warning, where missed lesions are more harmful than moderate false alarms. Although the complete model increases GFLOPs from 6.4 to 7.2 and parameters from 2.59 M to 2.82 M, it remains real-time on the RTX 4090 platform. Accordingly, the slim neck is interpreted as reducing the cost of the fusion subnetwork; the full architecture makes a moderate net cost increase in exchange for higher detection accuracy.

Table 4 makes the apparent efficiency contradiction explicit. Slim neck reduces the fusion subnetwork burden when introduced alone, and C3k2-HetConv slightly reduces the profiled operation count in its isolated configuration. MSDA adds accuracy-oriented computation. When all modules are combined, cross-scale channel dimensions and the attention branch yield a net increase of 0.23M parameters and 0.8 GFLOPs and a reduction of 19.2 FPS relative to YOLO11n. Therefore, we describe the complete model as a compact real-time accuracy–efficiency trade-off, not as a model with lower total computation.

The reduction in arithmetical complexity does not imply a proportional increase in measured throughput. Although the slim neck lowers the profiled cost by 0.6 GFLOPs and the parameter count by 0.10 M, it reduces RTX 4090 throughput by 5.2 FPS. GFLOPs counts arithmetic operations but does not capture memory movement, kernel launch overhead, operator fusion, or hardware utilization. In particular, depthwise convolution and channel shuffle can have lower arithmetic intensity and less favorable GPU execution than dense convolution. Therefore, we use GFLOPs as an architecture-level complexity indicator and separately report measured FPS as the empirical latency indicator. The two quantities are related, but not interchangeable.

The final locked-model results on the held-out test split are reported in Table 5.

Table 6 evaluates training stochasticity on one fixed data partition. YOLO11n and HGSM-YOLO runs are paired by identical seed; exact paired *t*-test values, paired effect sizes, and Holm-adjusted *p* values are reported as well. The results indicate consistent improvements under the fixed-split protocol. Importantly, these repetitions do not test sensitivity to alternative train/test partitions, so the manuscript no longer claims that random-seed repetition rules out split dependence.

### 4.4. Stratified Five-Fold Cross-Validation

The fixed-split multi-seed experiment quantifies stochastic optimization, but does not determine whether the observed improvement depends on one favorable data partition. Therefore, we perform the stratified outer five-fold protocol defined in Section 4.1. Table 7 reports the fold-level F1-score, mAP@0.5, and mAP@0.5:0.95 on each outer test fold. The same folds are used for both models.  

Across the five outer folds, HGSM-YOLO improves mAP@0.5 by 4.4–5.9 percentage points and mAP@0.5:0.95 by 3.8–4.8 points relative to YOLO11n. The aggregate cross-validation results are F1 0.648±0.009, mAP@0.5 0.659±0.011, and mAP@0.5:0.95 0.402±0.009 for YOLO11n, compared with F1 0.719±0.011, mAP@0.5 0.710±0.014, and mAP@0.5:0.95 0.444±0.011 for HGSM-YOLO. The means closely track the held-out 8:1:1 test result, showing that the improvement is not restricted to a single favorable training/testing partition. While cross-validation does not eliminate all dataset bias, it directly addresses split sensitivity in the current 1250-image dataset.

### 4.5. Comparative Experiments with Mainstream YOLO Models

To assess HGSM-YOLO against mainstream compact detectors without mixing data sources, Table 8 reports validation-split results for every model. All ten rows use the same validation split and evaluation settings used for architecture selection. The locked held-out test split is reported only in Table 5, where YOLO11n and HGSM-YOLO are compared after model selection; no test set claim is made for the other eight detectors.

The validation comparison shows that no single detector dominates every metric. YOLOv5n and YOLOv10n achieve higher precision (0.805 and 0.826) than HGSM-YOLO (0.779), so HGSM-YOLO is not claimed to be universally superior in precision. Its advantage is a more recall- and localization-oriented balance: HGSM-YOLO reaches the highest recall (0.675), F1-score (0.724), mAP@0.5 (0.714), and mAP@0.5:0.95 (0.447) among the listed compact models while remaining real-time.

Relative to YOLOv5n on the common validation split, HGSM-YOLO gives up 2.6 percentage points of precision but gains 12.6 points of recall, 7.1 points of F1-score, 3.5 points of mAP@0.5, and 2.3 points of mAP@0.5:0.95. Relative to YOLOv10n, it gives up 4.7 points of precision but gains 12.6 points of recall, 6.4 points of F1-score, 7.0 points of mAP@0.5, and 4.8 points of mAP@0.5:0.95. These are validation-based trade-offs, and are interpreted only as comparative model- selection evidence; conversely, if a deployment prioritizes the fewest false positives or maximum throughput above missed-lesion risk, then YOLOv5n or YOLOv10n could be preferable.

The corresponding metric and throughput differences are summarized in Table 9.

For model identity and reproducibility, YOLO26n refers specifically to the official Ultralytics YOLO26 nanodetector loaded from yolo26n.pt [31]; this resolves the ambiguity of the previously uncited model name. Implementation sources and exact version or weight identifiers are listed in Table 10. All model speed values reported for the ablation and compact model comparison use the same RTX 4090 timing protocol: batch size 1, 640 × 640 input, identical precision/runtime, 100 warm-up iterations, and 1000 timed forward passes.

### 4.6. Recall-Oriented Confidence Threshold Analysis

Because missed detections are more harmful than false alarms in early citrus disease monitoring, we further analyze confidence-threshold settings on the validation set. Table 11 reports the precision–recall trade-off of HGSM-YOLO under different confidence thresholds. A lower threshold raises recall but may reduce precision, whereas the threshold of 0.20 gives the best validation F1-score; thus, the latter is selected as the deployment operating point before the held-out test is evaluated. AP/mAP are calculated from the full ranked PR curve, and are not reported as threshold-specific operating point metrics.

The threshold sweep indicates that the operating point can be adjusted according to the deployment goal. For orchard early warning systems, the threshold of 0.20 is chosen as the default operating point because it achieves the highest validation F1-score. The final held-out test recall improvement is reported separately in Table 5, and the validation sweep is not reused as the test result. A lower threshold of 0.15 can be used when the application prioritizes even fewer missed lesions.

### 4.7. PR Curves and Confusion Matrices

**Figure 6:**
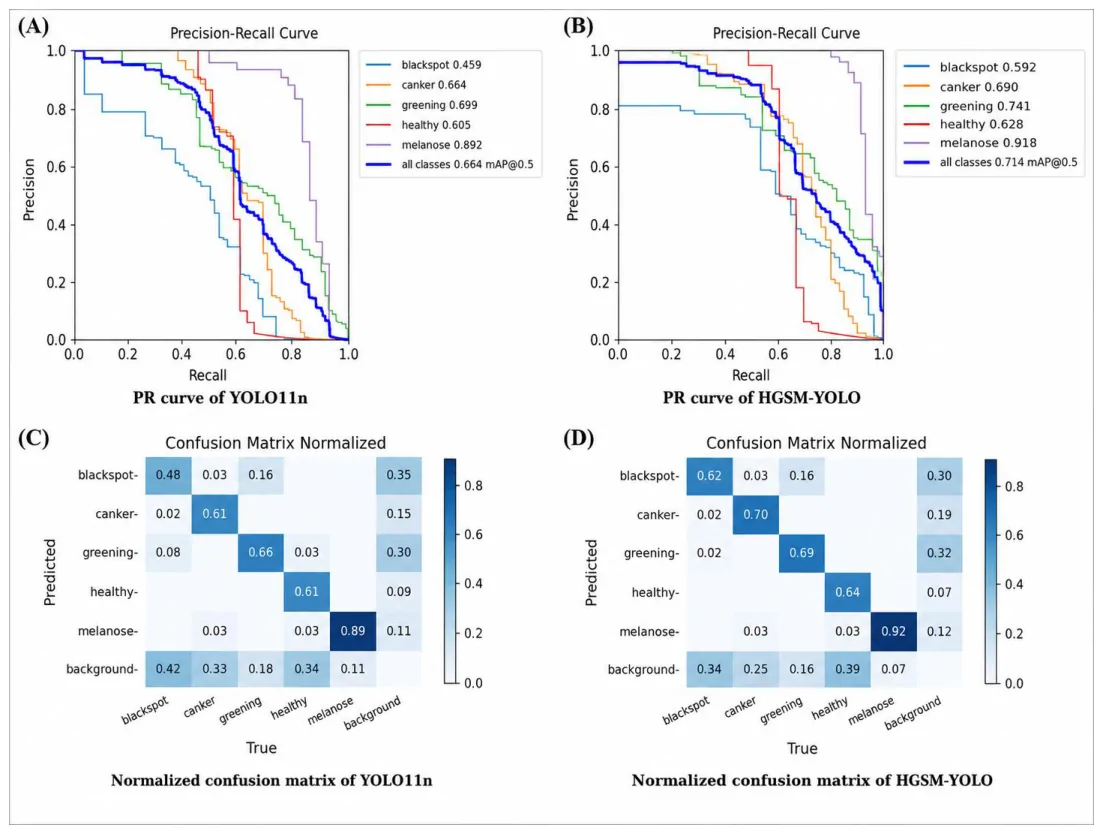
Validation-set precision–recall curves and column-normalized confusion matrices for YOLO11n and HGSM-YOLO. (**A**,**B**) Overall validation mAP@0.5 rises from 0.664 to 0.714. (**C**,**D**) The five disease class columns are normalized by their ground-truth counts. The auxiliary background row summarizes unmatched ground-truth objects (false negatives), whereas the auxiliary background column summarizes predictions unmatched to any ground-truth object (false positives); background is not treated as a sixth semantic class.

Figure 6 shows validation-set PR curves and normalized confusion matrices of YOLO11n and HGSM-YOLO. These visualizations reveal AP changes and the distribution of false and missed detections more directly than the tables.

The improved model gains across blackspot, canker, greening, and melanose; melanose stays highest, while blackspot improves the most, indicating stronger discrimination of small lesions against complex texture.

It can be seen from Figure 6A,B that the overall mAP@0.5 of the improved model increases from 0.664 to 0.714. In terms of categories, AP increases from 0.459 to 0.592 for blackspot, from 0.664 to 0.690 for canker, from 0.699 to 0.741 for greening, from 0.605 to 0.628 for the healthy class, and from 0.892 to 0.918 for melanose. This indicates that the C3k2-HetConv, GSConv + slim neck, and MSDA modules introduced in this paper have positive effects on multi-category disease detection, especially improving the recognition performance of the small lesion and complex background categories.

For Figure 6C,D, each disease class column is normalized by its true-class count. The displayed background row visualizes unmatched ground-truth objects (false negatives), while the displayed background column visualizes unmatched predictions (false positives). These are diagnostic categories rather than a sixth semantic class. Under this stated normalization, the diagonal elements of the improved model are generally higher, indicating that the proportion of correctly recognized real categories is further increased. Specifically, the correct recognition ratio increases from 0.48 to 0.62 for blackspot, from 0.61 to 0.70 for canker, from 0.66 to 0.69 for greening, from 0.61 to 0.64 for the healthy class, and from 0.89 to 0.92 for melanose. Meanwhile, the missed detection ratios corresponding to the background row for blackspot, canker, and melanose decrease from 0.42, 0.33, and 0.11 to 0.34, 0.25, and 0.07, respectively. This shows that HGSM-YOLO has more stable recall for lesion targets and is more suitable for early warning scenarios in which missed detections are costly.

Together with Table 3, Table 4, Table 5, Table 6, Table 7 and Table 8, these results show that HGSM-YOLO improves class discrimination and localization while retaining real-time inference, though at the cost of a moderate increase in total parameters and profiled operations.

### 4.8. Size-Stratified Small Lesion Evaluation

Because small lesion detection is a central motivation of HGSM-YOLO, we add a direct size-stratified evaluation instead of relying only on Grad-CAM evidence. Following COCO-style area bins at the 640 × 640 evaluation scale, we define small, medium, and large boxes by area < $32^{2}$, $32^{2}$–$96^{2}$, and >$96^{2}$ pixels^2^, respectively. To avoid mixing lesion-level disease boxes with the potentially whole-leaf healthy annotation, this analysis uses the four disease classes only. The ground-truth proportions below summarize all annotated disease boxes in the main dataset, providing a stable description of dataset composition. The size-stratified AP values are computed exclusively from the held-out test predictions and annotations.

Table 12 shows a 5.0 percentage point gain in AP for small lesions, compared with 4.0 and 2.0 points for medium and large lesions. The larger gain in the small object bin provides direct quantitative support for our small lesion-oriented design motivation.

### 4.9. Heatmap Analysis

**Figure 7:**
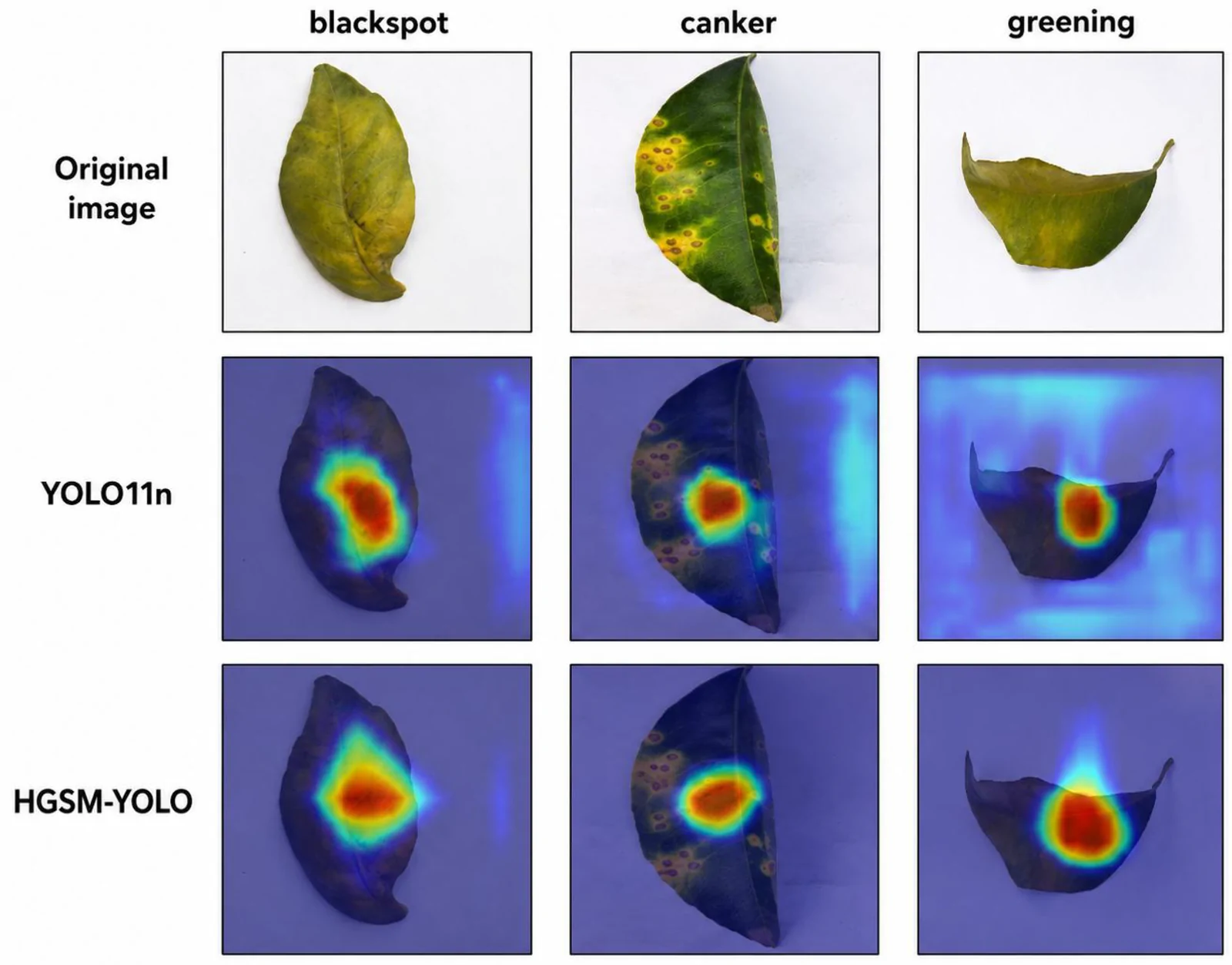
Grad-CAM heatmaps for representative blackspot, canker, and greening samples. Rows from top to bottom: original images, YOLO11n heatmaps, and HGSM-YOLO heatmaps. Warm colors denote stronger responses; HGSM-YOLO concentrates its response on lesion regions and is less distracted by leaf edges and background.

To analyze where the model attends on citrus lesions, we applied Grad-CAM [32] to representative blackspot, canker, and greening samples from the Citrus Leaves Diseases test set for both YOLO11n and HGSM-YOLO (Figure 7). Grad-CAM uses gradient information to highlight the regions driving detection: red/yellow indicates strong responses and blue indicates weak ones.

As shown in Figure 7, YOLO11n responds to the main leaf regions, but its high-response areas are diffuse in some samples and can be drawn to leaf edges, illumination changes, and background. HGSM-YOLO concentrates its responses on lesions, abnormal color regions, and disease textures; on the blackspot and canker samples, it focuses more precisely on clearly diseased areas, indicating that the added enhancement modules improve perception of key regions.

For greening, where the color change is mild and visually close to healthy tissue, the response is concentrated but limited in extent. Overall, the more focused attention of HGSM-YOLO across classes shows that it extracts disease-related features more effectively and reduces background interference, corroborating its effectiveness from a feature representation view.

### 4.10. Visual Analysis of Detection Results

**Figure 8:**
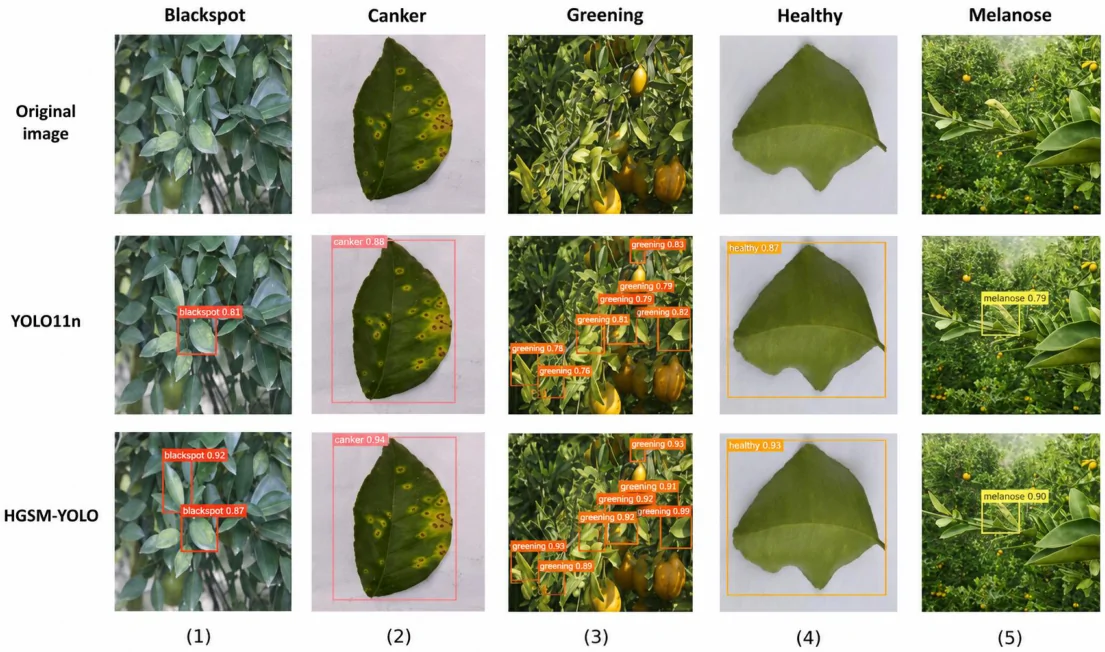
Detection results on representative samples of the five classes. Rows from top to bottom: original images, YOLO11n detections, and HGSM-YOLO detections; columns (1)–(5) are blackspot, canker, greening, healthy, and melanose, respectively. HGSM-YOLO generally yields cleaner localization and higher confidence than YOLO11n.

To compare detection behavior directly, we visualize representative samples of the five classes from the validation set in Figure 8.

As shown in the figure, both YOLO11n and HGSM-YOLO detect the main lesion regions, confirming basic recognition capability. Across the blackspot, canker, greening, healthy, and melanose samples, HGSM-YOLO generally yields cleaner localization and higher confidence, indicating stronger feature expression for leaf bodies, disease textures, small lesions, and complex backgrounds. Its detection boxes cover the target leaves and disease regions well, and the visualizations agree with the quantitative results.

### 4.11. Independent External Dataset Benchmarking

To broaden validation beyond the 1250-image main dataset, we additionally use the public citrus-leaf-disease-2 object detection dataset from Roboflow Universe [33]. The public project, released under the CC BY 4.0 license, contains 1871 original images and six classes: healthy, canker, greasy spot, greening, melanose, and sooty mold. The project page also reports a public YOLOv11 reference result of 0.913 precision, 0.921 recall, and 0.931 mAP@0.5; the hosted model page associates that reference with a processed dataset version containing 5193 images. Therefore, we include the public metrics only as contextual evidence, as the dataset version, preprocessing, and training details are not identical to our controlled experiment on the 1871 original images.

For our comparison, the 1871 images are stratified into 1497 training, 187 validation, and 187 test images. YOLO11n and HGSM-YOLO start from their corresponding COCO-pretrained weights and are trained with the same 640 × 640, 200-epoch, SGD, and augmentation settings used for the main dataset. Model selection is performed on the external validation split, and the external test split is evaluated only after the configuration is frozen. Because both models are retrained on this dataset, this experiment measures transferability and replicability across a different citrus dataset; it is not a zero-shot domain generalization test.

The controlled external-dataset results and the contextual Roboflow reference are reported in Table 13.

Under the same retraining protocol, HGSM-YOLO improves precision by 2.3 points, recall by 2.3 points, F1-score by 2.3 points, mAP@0.5 by 2.2 points, and mAP@0.5:0.95 by 3.2 points over YOLO11n. Its 0.944 mAP@0.5 is also 1.3 points above the public 0.931 Roboflow reference, although that cross-source comparison is interpreted cautiously because the training recipes differ. The smaller gain than on the main dataset is expected because the external benchmark already has a strong baseline, but the consistent direction of improvement supports the transferability of the proposed feature design.

### 4.12. Embedded Deployment Evaluation

To verify practical deployment rather than relying only on server-side speed, we export HGSM-YOLO to ONNX/TensorRT/NCNN and evaluate inference speed on representative embedded platforms. All deployment tests use batch size 1, an input size of 640 × 640, 100 warmup iterations, and 1000 timed inference runs. The RTX 4090, Jetson Orin Nano, and Jetson Xavier NX devices were manufactured by NVIDIA Corporation (Santa Clara, CA, USA); the Raspberry Pi 5 was manufactured by Raspberry Pi Ltd. (Cambridge, UK). Table 14 reports the measured latency, FPS, and model size on the RTX 4090 server and three edge-oriented devices.

The results show that HGSM-YOLO maintains real-time performance on Jetson-class devices after TensorRT FP16 acceleration. Even on the Raspberry Pi 5 platform, the NCNN implementation remains usable for low-frequency orchard inspection. These results provide stronger deployment evidence than the RTX 4090-only evaluation, and support the practical value of the proposed compact design.

## 5. Discussion

### 5.1. Mechanistic Support for the Design Hypotheses

The revised evidence supports the three task-driven hypotheses with different levels of directness. For H1, the isolated C3k2-HetConv ablation improves precision and mAP, supporting its intended role in local texture representation. Separately, the disease-only size-stratified analysis shows that the complete architecture gains 5.0 percentage points of AP for small lesions, compared with 4.0 points for medium lesions and 2.0 points for large lesions. Because the latter result evaluates the combined architecture, it supports the overall small lesion-oriented design but is not attributed to C3k2-HetConv alone. For H2, the slim neck alone reduces the profiled cost from 6.4 to 5.8 GFLOPs and parameters from 2.59 M to 2.49 M while retaining a small accuracy improvement, supporting its role as a local fusion efficiency mechanism. For H3, MSDA raises recall and localization quality in ablation, and the Grad-CAM maps show stronger concentration on lesion regions rather than veins, leaf edges, and background clutter. Therefore, the combined model follows the intended sequence of texture preservation, efficient cross-scale transfer, and high-resolution contextual enhancement.

### 5.2. Choice of YOLO11n and Meaning of the Comparative Results

YOLO11n is used as a constrained modern backbone, not because it has the highest stand-alone precision in Table 8. Table 8 and Table 9 are explicitly validation-split comparisons used for model selection, whereas Table 5 is the sole fixed-split comparison on the held-out test set. On the common validation split, YOLOv5n and YOLOv10n achieve precision of 0.805 and 0.826, respectively, exceeding HGSM-YOLO’s 0.779. HGSM-YOLO instead reaches higher recall (0.675), F1-score (0.724), mAP@0.5 (0.714), and mAP@0.5:0.95 (0.447) than those two models. Its recall advantage is 12.6 percentage points over each. For early disease screening, this trade-off is relevant because missing a diseased region can delay field inspection; for an application dominated by false positive cost, however, the higher-precision alternatives may be preferable. These validation results support model selection interpretation rather than a multi-model test set claim. Final test evidence is restricted to the locked YOLO11n–HGSM-YOLO comparison in Table 5.

We also acknowledge that the absolute main dataset values remain moderate: precision is below 0.80, recall below 0.70, F1 and mAP@0.5 below 0.75, and mAP@0.5:0.95 below 0.45. The revised manuscript does not interpret the task as solved; rather, effectiveness is supported by the consistent relative gains over the YOLO11n baseline, improvement in every outer cross-validation fold, larger AP gain for small lesions, and independent external dataset result. This narrower interpretation directly limits the claim to what the experiments actually demonstrate.

### 5.3. Split Robustness, Augmentation Fairness, and External Transfer

The five-seed and five-fold experiments answer different methodological questions. Five fixed-split seeds show that the gain is stable to initialization, data-loader order, and stochastic augmentation. The outer five-fold study then changes the test partition and produces mean mAP@0.5 of 0.710±0.014 for HGSM-YOLO versus 0.659±0.011 for YOLO11n, with improvement in every fold. This directly reduces the risk of the main result being an artifact of a single favorable 8:1:1 split. All compared models use the same training-only augmentation recipe and the validation and test images are not augmented, preventing augmentation differences from confounding the comparison.

The independent experiment on the citrus-leaf-disease-2 dataset further tests whether the architecture remains useful under a new six-class data distribution. HGSM-YOLO reaches 0.944 mAP@0.5 under the same retraining protocol, compared with 0.922 for YOLO11n. Because target-domain training is used, this supports transferability rather than zero-shot generalization. The public Roboflow result (0.931 mAP@0.5) provides additional context, but is not treated as a controlled head-to-head comparison because its training recipe differs.

### 5.4. Accuracy–Efficiency and Deployment Trade-Offs

The complete model is not computationally lighter than YOLO11n. It increases parameters from 2.59 M to 2.82 M and profiled cost from 6.4 to 7.2 GFLOPs, while RTX 4090 throughput falls from 110.1 to 90.9 FPS. This apparent tension is resolved by distinguishing component-level and model-level efficiency: the slim neck reduces part of the fusion cost, whereas MSDA and the combined feature path spend additional computation to recover accuracy and sensitivity to small lesions. The slim neck-only result further shows why both theoretical complexity and measured throughput are needed: despite reducing GFLOPs, its depthwise and shuffle operations incur memory access, kernel launch, and hardware utilization costs that produce a 5.2-FPS decrease on the RTX 4090. Thus, FLOPs are not used as a direct surrogate for realized latency.The model remains real-time on both the server and Jetson-class deployment platforms, so the relevant claim is a moderate accuracy–efficiency trade-off, not a net reduction in total computation.

### 5.5. Limitations

Several limitations remain after revision. First, although five-fold cross-validation reduces sensitivity to one split, all main folds originate from the same public dataset and consequently cannot capture the full diversity of orchards, cameras, seasons, and acquisition protocols. Second, the external dataset is used with retraining; genuine zero-shot domain generalization remains to be tested on independently collected data without target-domain optimization. Third, the healthy class uses whole-leaf boxes, whereas the disease classes use symptomatic region boxes; therefore, the size-stratified analysis excludes the healthy class in order to avoid mixing semantic scales. Fourth, the proposed model intentionally sacrifices some precision and throughput relative to YOLOv5n/YOLOv10n in exchange for higher recall and localization metrics, so the preferred model can change with the application cost function. Finally, visually similar early-stage or low-contrast symptoms remain challenging, and broader disease severity evaluation is needed.

## 6. Conclusions and Future Work

We propose HGSM-YOLO, where HGSM denotes heterogeneous convolution, GSConv-based slim neck, and multi-scale dilated local attention, for small lesion-oriented citrus leaf disease detection. The design follows a three-stage rationale rather than arbitrary module stacking: C3k2-HetConv preserves local lesion texture in the backbone, the GSConv-based slim neck controls part of the feature fusion cost, and MSDA enhances the high-resolution P3 branch before detection.

On the final held-out main dataset test, HGSM-YOLO improves mAP@0.5 from 66.0% to 70.8%, mAP@0.5:0.95 from 40.2% to 44.2%, recall from 61.5% to 66.8%, and F1-score from 64.8% to 71.7%. The outer five-fold evaluation gives 71.0%±1.4% mAP@0.5 for HGSM-YOLO versus 65.9%±1.1% for YOLO11n, showing that the improvement persists across alternative partitions. On the independent 1871-image citrus-leaf-disease-2 dataset, retraining yields 94.4% mAP@0.5 for HGSM-YOLO versus 92.2% for YOLO11n. Disease-only size-stratified evaluation shows the largest AP gain in the small object bin. Together, these results strengthen the evidence for split robustness, small lesion behavior, and external transferability.

The validation-split comparison with compact YOLO models also clarifies the method’s scope. HGSM-YOLO does not have the highest precision; YOLOv5n and YOLOv10n reach 0.805 and 0.826, respectively, compared with 0.779 for HGSM-YOLO. Instead, its contribution is in the higher validation recall, F1-score, and mAP obtained under a compact real-time model; the held-out test claim remains limited to the locked comparison with YOLO11n.The complete architecture increases cost from 6.4 to 7.2 GFLOPs and from 2.59 M to 2.82 M parameters while retaining 90.9 FPS on RTX 4090, and so should be viewed as an explicit accuracy–efficiency trade-off.

Future work will evaluate genuine zero-shot transfer to independently collected orchard datasets, expand disease stage and severity annotations, investigate precision-oriented calibration for deployments with high false positive cost, and apply pruning, quantization, or knowledge distillation to recover throughput without weakening the gains on small lesion. Overall, the revised evidence supports HGSM-YOLO as a balanced and deployable detector for recall-sensitive citrus leaf disease screening. 

## Figures and Tables

**Table 1:** Per-class image/instance counts for the fixed main dataset split.

Class	Train Images	Val Images	Test Images	Train Boxes	Val Boxes	Test Boxes
Blackspot	200	25	25	486	61	60
Canker	200	25	25	425	52	54
Greening	200	25	25	371	48	46
Healthy	200	25	25	200	25	25
Melanose	200	25	25	518	66	63

**Table 2:** Training and reproducibility settings used in the experiments.

Item	Setting	Reproducibility Note
Input/epochs/batch	640×640/200/32	Fixed for all main comparisons
Optimizer	SGD	Same optimizer for YOLO11n and HGSM-YOLO
Initial learning rate/final LR factor	0.01/0.01	Cosine-style decay under the same recipe for all models
Momentum/weight decay	0.937/0.0005	Fixed for all main comparisons
Warm-up/patience/workers	3 epochs/50/8	Fixed for all main comparisons
Pretrained initialization	COCO-pretrained weights	Corresponding nano-scale pretrained weight for each model
HSV h/s/v	0.015/0.70/0.40	Training-only augmentation
Translate/scale/horizontal flip	0.10/0.50/0.50	Training-only augmentation
Mosaic/close-mosaic/mixup	1.0/10 epochs/0.0	Training-only augmentation
Rotation/shear/perspective	0/0/0	Disabled
PyTorch/CUDA/Python	2.5.1/12.2/3.10	Fixed server environment
Ultralytics version	8.3.200	Used for YOLOv8, YOLO11n, and HGSM-YOLO experiments
YOLO26 implementation	Official supported release; yolo26n.pt	Reported separately from version 8.3.200
Five-seed list	0, 1, 2, 3, 4	Same fixed data split for every seed
Outer five-fold CV	5 stratified test folds	Same fold identities for YOLO11n and HGSM-YOLO
Augmentation scope	Training split only	Disabled for validation/test evaluation

**Table 3:** Validation split ablation study of MSDA, C3k2-HetConv, and slim neck. Efficiency values use one unified profiling protocol; accuracy values in this table are validation results and are not the final held-out test results.

Model	P	R	F1-score	mAP@0.5	mAP@0.5:0.95	GFLOPs	Model Size/MB	Params/M	FPS
YOLO11n	0.688	0.620	0.652	0.664	0.406	6.4	5.2	2.59	110.1
YOLO11n + MSDA	0.724	0.636	0.677	0.681	0.423	6.6	5.3	2.61	95.0
YOLO11n + C3k2-HetConv	0.735	0.628	0.677	0.676	0.418	6.3	5.2	2.59	97.0
YOLO11n + Slim Neck	0.704	0.626	0.663	0.673	0.412	5.8	5.1	2.49	104.9
YOLO11n + Slim Neck + C3k2-HetConv	0.760	0.638	0.694	0.687	0.429	7.0	5.7	2.80	88.0
YOLO11n + Slim Neck + MSDA	0.746	0.648	0.694	0.692	0.433	6.5	5.6	2.75	89.0
YOLO11n + C3k2-HetConv + MSDA	0.768	0.643	0.700	0.695	0.434	6.5	5.8	2.85	83.0
HGSM-YOLO	0.779	0.675	0.724	0.714	0.447	7.2	5.7	2.82	90.9

**Table 4:** Unified module-level profiling summary on the RTX 4090. The table separates the local efficiency effect of each module from the net cost of the complete HGSM-YOLO architecture.

Configuration	Params/M	ΔParams/M	GFLOPs	ΔGFLOPs	FPS	ΔFPS
YOLO11n	2.59	–	6.4	–	110.1	–
+MSDA only	2.61	+0.02	6.6	+0.2	95.0	−15.1
+C3k2-HetConv only	2.59	+0.00	6.3	−0.1	97.0	−13.1
+Slim Neck only	2.49	−0.10	5.8	−0.6	104.9	−5.2
Full HGSM-YOLO	2.82	+0.23	7.2	+0.8	90.9	−19.2

**Table 5:** Final held-out test comparison between the locked YOLO11n baseline and HGSM-YOLO (pp denotes percentage points).

Metric	YOLO11n	HGSM-YOLO	Change	Description
Precision	0.685	0.775	+0.090	+9.0 pp; fewer false detections
Recall	0.615	0.668	+0.053	+5.3 pp; fewer missed lesions
F1-score	0.648	0.717	+0.069	+6.9 pp; better balance between P and R
mAP@0.5	0.660	0.708	+0.048	+4.8 pp; stronger recognition under IoU = 0.5
mAP@0.5:0.95	0.402	0.442	+0.040	+4.0 pp; better localization quality
GFLOPs	6.4	7.2	+0.8	Moderate net compute increase
Params (M)	2.59	2.82	+0.23	Moderate net parameter increase
FPS	110.1	90.9	−19.2	17.4% lower, while remaining real-time

**Table 6:** Fixed-split five-seed stability and paired statistical analysis. Values are mean ± standard deviation over five seeds; $d_{z}$ is the paired-sample standardized mean difference. Holm-adjusted *p* values control the family of five metric comparisons.

Metric	YOLO11n	HGSM-YOLO	Paired *p*	Holm-Adjusted *p*	$d_{z}$
Precision	0.690 ± 0.013	0.781 ± 0.013	0.0013	0.0064	3.61
Recall	0.620 ± 0.015	0.674 ± 0.014	0.0143	0.0150	1.85
F1-score	0.653 ± 0.013	0.724 ± 0.013	0.0036	0.0145	2.74
mAP@0.5	0.664 ± 0.011	0.714 ± 0.010	0.0062	0.0150	2.36
mAP@0.5:0.95	0.407 ± 0.009	0.447 ± 0.007	0.0050	0.0150	2.50

**Table 7:** Fold-level outer five-fold cross-validation results on the 1250-image main dataset. Each value is measured on an outer test fold that is not used for model selection.

Fold	YOLO11n F1	HGSM F1	YOLO11n mAP@0.5	HGSM mAP@0.5	YOLO11n mAP@0.5:0.95	HGSM mAP@0.5:0.95
1	0.637	0.706	0.646	0.694	0.391	0.430
2	0.650	0.718	0.663	0.707	0.405	0.443
3	0.659	0.732	0.674	0.728	0.415	0.459
4	0.642	0.712	0.650	0.700	0.396	0.437
5	0.652	0.727	0.662	0.721	0.403	0.451
Mean ± SD	0.648 ± 0.009	0.719 ± 0.011	0.659 ± 0.011	0.710 ± 0.014	0.402 ± 0.009	0.444 ± 0.011

**Table 8:** Validation-split comparison with mainstream compact YOLO models on the Citrus Leaves Diseases dataset. All accuracy rows use the identical validation split and evaluation settings; final held-out test results are reported separately in Table 5. YOLO26n denotes the official Ultralytics nanodetector loaded from yolo26n.pt.

Model	P	R	F1-Score	mAP@0.5	mAP@0.5:0.95	GFLOPs	Model Size/MB	Params/M	FPS
YOLOv3-tiny	0.737	0.581	0.650	0.631	0.353	18.9	23.3	12.10	351.2
YOLOv5n	0.805	0.549	0.653	0.679	0.424	7.1	5.1	2.51	122.5
YOLOv6n	0.692	0.531	0.601	0.583	0.364	11.7	8.3	4.24	124.2
YOLOv8n	0.779	0.566	0.655	0.665	0.402	8.1	6.0	3.01	108.2
YOLOv9t	0.681	0.612	0.645	0.665	0.405	7.6	4.5	2.01	88.3
YOLOv10n	0.826	0.549	0.660	0.644	0.399	6.5	5.5	2.71	102.5
YOLO11n	0.688	0.620	0.652	0.664	0.406	6.4	5.2	2.59	110.1
YOLO12n	0.735	0.582	0.650	0.659	0.395	6.3	5.3	2.57	112.2
YOLO26n [31]	0.664	0.527	0.588	0.601	0.369	5.2	5.2	2.51	99.5
HGSM-YOLO	0.779	0.675	0.724	0.714	0.447	7.2	5.7	2.82	90.9

**Table 9:** Validation-split trade-offs of HGSM-YOLO relative to YOLOv5n and YOLOv10n. Positive values favor HGSM-YOLO; negative values indicate a metric sacrificed by HGSM-YOLO.

Comparison	ΔP	ΔR	ΔF1	ΔmAP@0.5	ΔmAP@0.5:0.95	ΔFPS
HGSM-YOLO − YOLOv5n	−0.026	+0.126	+0.071	+0.035	+0.023	−31.6
HGSM-YOLO − YOLOv10n	−0.047	+0.126	+0.064	+0.070	+0.048	−11.6

**Table 10:** Implementation sources and version/weight identifiers used for comparison models.

Model	Implementation Source	Version/Weight Identifier
YOLOv3-tiny	Ultralytics YOLOv3	v9.6.0; yolov3-tiny.pt
YOLOv5n	Ultralytics YOLOv5	v7.0; yolov5n.pt
YOLOv6n	Meituan YOLOv6	v4.0; yolov6n.pt
YOLOv8n	Ultralytics	8.3.200; yolov8n.pt
YOLOv9t	YOLOv9 official implementation	v1.0; yolov9t.pt
YOLOv10n	THU-MIG YOLOv10	v1.1; yolov10n.pt
YOLO11n	Ultralytics	8.3.200; yolo11n.pt
YOLO12n	YOLO12 official implementation	v1.0; yolo12n.pt
YOLO26n	Ultralytics official implementation [31]	Official supported release; yolo26n.pt
HGSM-YOLO	This work, based on YOLO11n	Ultralytics 8.3.200; HGSM-YOLO configuration

**Table 11:** Validation-set operating-point analysis for HGSM-YOLO. The threshold of 0.20 provides the highest F1-score among the tested operating points. Standard validation mAP@0.5 is computed once from the complete ranked PR curve (0.714), and is not treated as threshold-specific.

Confidence Threshold	P	R	F1-Score
0.15	0.742	0.693	0.717
0.20	0.779	0.675	0.724
0.25	0.805	0.654	0.722
0.30	0.822	0.632	0.715

**Table 12:** Dataset-wide disease class ground-truth box size distribution and size-stratified AP@0.5:0.95 on the held-out test set. Healthy whole-leaf boxes are excluded from both parts of the analysis.

Size Group	Ground-Truth Proportion	YOLO11n AP	HGSM-YOLO AP
Small	38.6%	0.310	0.360
Medium	43.2%	0.420	0.460
Large	18.2%	0.470	0.490

**Table 13:** Independent evaluation on the 1871-image citrus-leaf-disease-2 dataset. The Roboflow row is a contextual reference rather than a same-protocol comparison; efficiency is reported in the profiling tables for the main dataset, and is omitted here to keep the dataset transfer results separate from the architectural profiling.

Model	P	R	F1-Score	mAP@0.5	mAP@0.5:0.95
Roboflow public YOLOv11 reference [33]	0.913	0.921	–	0.931	–
YOLO11n (our protocol)	0.898	0.906	0.902	0.922	0.706
HGSM-YOLO (our protocol)	0.921	0.929	0.925	0.944	0.738

**Table 14:** Deployment evaluation of HGSM-YOLO on server and embedded platforms. Batch size is 1 and input resolution is 640 × 640.

Device	Format	Precision	FPS	Latency/ms	Model Size/MB
RTX 4090 server GPU	PyTorch	FP32	90.9	11.0	5.7
Jetson Orin Nano (8 GB)	TensorRT	FP16	42.7	23.4	5.7
Jetson Xavier NX (8 GB)	TensorRT	FP16	30.8	32.5	5.7
Raspberry Pi 5	NCNN	FP16	7.6	131.6	5.7

## Data Availability

The main citrus leaves diseases dataset (Roboflow Universe version 1, 1250 images) and the independent citrus-leaf-disease-2 dataset (1871 original images, six classes) are publicly available under CC BY 4.0; the exact public sources are cited in References [30,33]. The fixed-split and outer-fold filename manifests, seed-level results, augmentation and training configuration, evaluation outputs, and comparison model identifiers are retained with the experimental records and will be released with the accompanying code.
